# The Relationship of Dental Aesthetic Index with Dental Appearance, Smile and Desire for Orthodontic Correction

**DOI:** 10.5005/jp-journals-10005-1023

**Published:** 2009-08-26

**Authors:** Ullal Anand Nayak, Jasmin Winnier, Rupesh S

**Affiliations:** 1 Professor, Department of Pedodontics, Modern Dental College and Research Center, Indore, Madhya Pradesh, India; 2 Senior Lecturer, Department of Pedodontics, Dr DY Patil Dental College and Hospital, Navi Mumbai, Mumbai Maharashtra, India; 3 Senior Lecturer, Department of Pedodontics, Pushpagiri College of Dental Sciences, Perunduruthy, Kerala, India

**Keywords:** Dental aesthetic index, dental appearance, desire for orthodontic correction.

## Abstract

*Objective:* The purpose of this study was to determine the relationship between dental aesthetic index (DAI) and self – satisfaction with dental appearance, smile and desire for orthodontic care.

*Methods:* A survey of 103 school children, 51 boys and 52 girls was carried out in Annamalai Nagar, Chidambaram. The subjects were interviewed using a questionnaire consisting of questions concerning smile, dental appearance and desire for orthodontic treatment. They were also assessed using the dental aesthetic index.

*Results:* Statistically significant correlations were between subjective assessments of dental appearance and DAI (P = 0.042) and need for orthodontic treatment and DAI (P = 0.045). The strongest correlations were found between DAI and comparative evaluations of dental appearance (P < 0.005).

*Conclusion:* This study has shown significant correlations between DAI and subjective perceptions of dental appearance.

## INTRODUCTION

The traditional opinions regarding the major benefits of orthodontic treatment have been challenged. The importance of orthodontic care in the prevention of caries, periodontal disease and temporomandibular joint disorders are beginning to be doubted. Orthodontic treatment is often carried out for aesthetic rather than functional considerations, since it is assumed that failure to meet social norms for dental aesthetics will have negative psychosocial effects and these effects may well-exceed the biological problems.^1^ Dentists predict that psychosocial component of malocclusion will continue to be one of the strongest motivator for orthodontic treatment.^2^

The measurement of malocclusion as a public health problem is extremely difficult since most orthodontic treatment is undertaken for aesthetic reasons and it is very difficult to estimate the extent to which malposed teeth or dentofacial malrelation constitute to a psychological hazard.^2^ Malocclusion has proved to be a difficult entity to define since individual perceptions of what constitutes a malocclusion problem differs widely.^3^

Orthodontic indices were developed in the late 1960’ and the early 70s in order to assist professionals in categorizing malocclusion according to the level of treatment need. These indices include Salzman’ handicapping malocclusion assessment record (HAMAR),^4^ Summer’ occlusal index (OI)^5^ and Graingers treatment priority index (TPI).^6^

The dental aesthetic index (DAI)^2^ and the index of orthodontic treatment need (IOTN)^7^,^8^ are both used for assessment of malocclusion and determination of treatment need. The DAI, however, is a cross-cultural index.^6^

In order to assess whether DAI provides reliable information regarding the subjects’ psychosocial desire for treatment, an interview schedule was used. Ten items were included in the interview that asked for self-evaluation of dental appearance, satisfaction with smile and desire for orthodontic care.^2^

With this background, the present study was conducted with the following aims and objectives:

1. To assess the relationship between DAI and selfsatisfaction with smile.
2. To assess the relationship between DAI and selfsatisfaction with dental appearance.
3. To assess the relationship between DAI and selfassessment of the relative appearance of teeth by the subjects as compared to their face.
4. To assess the relationship between DAI and selfassessment of dental appearance compared to classmates and friends.
5. To assess the relationship between DAI and desire to undergo orthodontic treatment.

## SUBJECTS AND METHODS

103 subjects (51 boys and 52 girls) who satisfied the following criteria were selected for the study.

Inclusion criteria:

The subject was then assessed by the DAI. All the 10 components were measured.

Exclusion criteria:

1. Children with mental or physical impairment.
2. Previous history of or currently undergoing orthodontic treatment.
3. Subjects who had decided about orthodontic treatment at the time of study.

Informed consent from relevant school authorities and verbal consent from participants were obtained. A single examiner examined all the subjects. The examination was carried out in the subjects’ school, during daytime under natural light with the help of Boley gauge, a sharp pencil, tongue blade and a millimeter ruler. The accepted methods of infection control were maintained. The examiner asked the following questions to each subject. The need for privacy and confidentiality was stressed. Each subject was interviewed privately before being examined.

1. Do you have a pleasant smile?       Yes/No
2. How much do you like the appearance of your smile?
  - Very much
  - Quite a bit
  - Not much
  - Not at all
3. Do you like the way your teeth look?       Yes/No
4. How much do you like the way your teeth look?
  - Very much
  - Quite a bit
  - Not much
  - Not at all
5. Are your front teeth straight?       Yes/No
6. How would you consider your teeth as compared to your entire face?
  - One of the nicest features of your face
  - Better than average feature of your face
  - Below average feature of your face
  - One of the poorest features of your face
7. Are your teeth good looking?       Yes/No
8. Compared to your classmates and friends how do you think your teeth look?
  - Among the nicest
  - Better than average
  - Below average
  - Among the worst
9. Do your teeth need straightening?       Yes/No
10. If it were possible would you want to wear braces to straighten your teeth?
  - Definitely No
  - Probably No
  - Probably Yes
  - Definitely Yes

The subject was then assessed by the DAI. All the 10 components were measured.

Components of the DAI regression equation and their actual and rounded regression coefficients (weights):

**Table 1:** 

*Number*		*DAI components*	*Actual weights*	*Rounded weights*
1		Number of missing visible teeth (Incisors, canines and premolars) in the maxillary and mandibular arches.	5.76	6
2		Assessment of crowding in the incisal segments: 0 = No segment crowded, 1 = One segment crowded, 2 = Two segments crowded.	1.15	1
3		Assessment of spacing in the incisal segments: 0 = No segment spaced, 1 = One segment spaced, 2 = Two segments spaced.	1.31	1
4		Measurement of any midline diastema in mm	3.13	3
5		Largest anterior irregularity on the maxilla in mm	1.34	1
6		Largest anterior irregularity on the mandible in mm	0.75	1
7		Measurement of anterior maxillary overjet in mm	1.62	2
8		Measurement of anterior mandibular overjet in mm	3.68	4
9		Measurement of vertical anterior openbite in mm	3.69	4
10		Assessment of anteroposterior molar relation: Largest deviation from normal either left or right, 0 = Normal, 1 = Half cusp either mesial or distal, 2 = One full cusp either mesial or distal.	2.69	3
		Constant	13.36	13

The measured components of the DAI were multiplied by the regression coefficient (weights), the products are added, and the constant number 13 was added to the total to give the final DAI score.

The treatment need according to the scores is divided into the following categories: 9

- *25 and below:* Normal or minor malocclusion with no or slight treatment need.
- *
26 to 30:* Definite malocclusion; elective treatment.
- *
30 to 35:* Severe malocclusion; treatment highly desirable.
- *36 and more:* Handicapping malocclusion; treatment mandatory.

## RESULTS

Of the 103 subjects examined only 20% definitely needed orthodontic treatment according to the DAI.

In response to the question regarding satisfaction with smile, 83.5% of subjects were satisfied with the appearance of their smile, 63.1% stated that they liked the way their teeth looked. 70.8% felt that their teeth were better than average or one of the nicest features of their face. 66% felt that compared to their classmates and friends they had a better than average or one of the nicest dentitions and 35% responded that they would definitely wear braces if it would improve their dental appearance (Table 1).

Statistically there was no agreement present between smile and dental aesthetic index P = 0.269 (Tables 2 and 2A).

However, there was a minimal but statistically significant agreement between the index and the patients' selfsatisfaction with their dental appearance P < 0.05 (Tables 3 and 3A).

The relationship between DAI and the self-assessment of relative appearance of teeth by the subjects as compared to their face was highly statistically significant P < 0.005 (Tables 4 and 4A).

**Table:** TABLE 1: Questionnaire analysis

Score		Q2		Q4		Q6		Q8		Q10	
1		44.7%		30.1%		25.2%		20.4%		33%
2		38.8%		33%		45.6%		45.6%		11.7%
3		13.6%		29.1%		22.3%		32%		20.4%
4		2.9%		7.8%		6.8%		1.9%		35%

**Table 2:** TABLE 2: The relationship between DAI and Q2 (How much do you like the appearance of your smile?)

					*Smile scores*															
					*1 *			*2 *			*3 *			*4 *			*Total*
**DAI scores**		1: No treatment need			29.1%			22.3%			7.8%			.0%			59.2%
		2: Elective treatment			9.7%			6.8%			1.9%			1.9%			20.4%
		3: Treatment desirable			2.9%			5.8%			3.9%			.0%			12.6%
		4: Treatment mandatory			2.9%			3.9%			.0%			1.0%			7.8%
**Total**					**44.7%**			**38.8%**			**13.6%**			**2.9%**			**100%**

**Table 2A:** TABLE 2A: Analysis

		*Value*		*Approx. sig*
Measure of agreement Kappa		0.070		0.269

**Table 3:** TABLE 3: The relationship between DAI and Q4 (How much do you like the way your teeth look?)

					*Appearance of teeth scores*															
					*1 *			*2 *			*3 *			*4 *			*Total*
**DAI scores**		1: No treatment need			20.4%			19.4%			16.5%			2.9%			59.2%
		2: Elective treatment			6.8%			9.7%			1.9%			1.9%			20.4%
		3: Treatment desirable			2.9%			2.9%			4.9%			1.9%			12.6%
		4: Treatment mandatory			.0%			1.0%			5.8%			1.0%			7.8%
**Total**					**30.1%**			**33.0%**			**29.1%**			**7.8%**			**100%**

**Table 3A:** TABLE 3A: Analysis

		*Value*		*Approx. sig*
Measure of agreement Kappa		0.112		0.042

**Table 4:** TABLE 4: The relationship between DAI and Q6 (How would you consider your teeth as compared to your entire face?)

					*Relative appearance of teeth*															
					*1 *			*2 *			*3 *			*4 *			*Total*
**DAI scores**		1: No treatment need			20.4%			25.2%			11.7%			1.9%			59.2%
		2: Elective treatment			1.9%			14.6%			2.9%			1.0%			20.4%
		3: Treatment desirable			2.9%			2.9%			3.9%			2.9%			12.6%
		4: Treatment mandatory			.0%			2.9%			3.9%			1.0%			7.8%
**Total**					**25.2%**			**45.6%**			**22.3%**			**6.8%**			**100%**

**Table 4A:** TABLE 4A: Analysis

		*Value*		*Approx. sig*
Measure of agreement Kappa		0.169		0.002

**Table 5:** TABLE 5: The relationship between DAI and Q8 (Compared to your classmates and friends how do you think your teeth look?)

					*Comparative assessment of dental appearance*															
					*1 *			*2 *			*3 *			*4 *			*Total*
**DAI scores**		1: No treatment need			16.5%			31.1%			11.7%			.0%			59.2%
		2: Elective treatment			1.0%			11.7%			6.8%			1.0%			20.4%
		3: Treatment desirable			2.9%			1.9%			7.8%			.0%			12.6%
		4: Treatment mandatory			.0%			1.0%			5.8%			1.0%			7.8%
**Total**					**20.4%**			**45.6%**			**32.0%**			**1.9%**			**100%**

**Table 5A:** TABLE 5A: Analysis

		*Value*		*Approx. sig*
Measure of agreement Kappa		0.152		0.003

**Table 6:** TABLE 6: The relationship between DAI and Q 10 (If it were possible would you want to wear braces to straighten your teeth?)

					*Desire for orthodontic care*															
					*1 *			*2 *			*3 *			*4 *			*Total*
**DAI scores**		1: No treatment need			25.2%			7.8%			12.6%			13.6%			59.2%
		2: Elective treatment			4.9%			2.9%			3.9%			8.7%			20.4%
		3: Treatment desirable			2.9%			1.0%			1.0%			7.8%			12.6%
		4: Treatment mandatory			.0%			.0%			2.9%			4.9%			7.8%
**Total**					**33.0%**			**11.7%**			**20.4%**			**35.0%**			**100%**

**Table 6A:** TABLE 6A: Analysis

		*Value*		*Approx. sig*
Measure of agreement Kappa		0.139		0.045

There relationship between DAI and the self-assessment of dental appearance as compared to classmates and friends was highly statistically significant P < 0.005 (Tables 5 and 5A).

There was also a statistically significant relationship present between the index and the willingness of the subject to accept orthodontic treatment P < 0.05 (Tables 6 and 6A).

## DISCUSSION

The DAI was developed by Cons, Jenny and Kohout in 1987.^2^ It is a relatively simple index; it can be obtained intraorally, without the use of radiographs in about two minutes. The reliability and validity of DAI has been well documented in various studies.^1^,^9^,^10^ It has been accepted by the WHO as a cross-cultural index.^6^ It was integrated into the items of International collaboration study of oral health outcomes by the WHO in 1989.^11^,^12^ Although DAI is easy to use, there is lack of assessment traits such as buccal cross bite, open bite, centerline discrepancy and deep bite.^11^ Though these may not be important from a dental aesthetic point of view, they could affect the need for orthodontic treatment.^9^

The cut off point of any treatment need index is the value below which the severity of malocclusion is so minor that there is no definite need for treatment and all values above that point indicate malocclusion for which treatment is mandatory. The recommended treatment cut off point for Dental Aesthetic Index is 31.^13^ In the present study, a majority of the patients were below the cut off point, delineating the group of children who were definitely in need of orthodontic treatment.

The DAI has been compared with other treatment need indices in various studies.^1^,^9^-^11^,^13^-^15^ It has been used in an epidemiological assessment of malocclusion in Japan^12^ and in evaluation of outcomes of orthodontic treatment.^16^ In addition, it has also been used along with structured questionnaires regarding appearance, biting/chewing, speech and orthodontic treatment need.^11^,^6^

However, the patient’s opinions regarding orthodontic treatment need cannot be underestimated, as it is the patient who receives treatment and needs to gain satisfaction from improved aesthetics and function or both.^11^ Also it is known that the parent or the patients’ concerns of orthodontic treatment need, do not always agree with professional evaluations of the same.^6^,^11^ In addition, orthodontic treatment is primarily influenced by demand and not always by need.^17^ Hence, in the present study, the relationship of Dental Aesthetic Index with selected questions, which reflect the psychosocial need for orthodontic treatment, was assessed.

Adolescence is the age when increase in awareness and facial aesthetics takes priority and adolescent children have a tendency to compare themselves with peers, models, etc. The age group of subjects in the present study was 13 to 16 years. Hence, the questionnaire used in our study was aimed at children in this adolescent age group to assess their awareness of dental appearance and to evaluate their relationship with an objective measure of aesthetics (DAI).

The present study revealed that the association between DAI and self-satisfaction with smile was not significant. This, however, is in contrast to the results of Cons et al.^2^ Thus, in the present population, dissatisfaction with smile cannot be taken as an indicator of need for orthodontic treatment.

There was a weak but significant correlation between DAI and self-satisfaction with dental appearance, which is in agreement with the study by Onyeaso et al,^6^ but is in contrast to the study by Yeh et al,^11^ which showed no significant relationship.

The strongest correlations in this study were found between DAI and the subjects’ self-assessment of relative appearance of teeth as compared to their face and the selfassessment of dental appearance compared to their classmates and friends, corroborates with the correlation found previously.^2^

The analysis also revealed a weak but statistically significant relationship between DAI and desire for orthodontic care.

Hamdan AM (2004)^17^ reported that twice as many females presented for orthodontic consultation than males. Holmes (1992) suggested that greater number of females perceived themselves as having less attractive dentitions than males despite any objective evidence to support this view. Also 75% of subjects who seek orthodontic treatment do so for aesthetic reasons and girls are more likely to recognize dental irregularities and place more importance on this than boys.^18^ However, in the present study, of the subjects who were not satisfied with the appearance of their teeth 15 were male and 23 were female subjects, though this difference was not statistically significant. Almost equal number of boys and girls stated that they would be willing to wear braces if it would improve their dental appearance.

Desire for treatment has been noted to be more frequent than dissatisfaction with appearance.^18^-^21^ Similarly, in the present study also, the need for orthodontic treatment as assessed by DAI was 20.4% where as the demand was 35%. Elham (2004)^22^ found 49% demand for orthodontic treatment among school children in North Jordan. The relatively high number of persons expressing desire for treatment may reflect a professional trust or a basic general faith in service.^18^,^19^,^23^,^24^ Such an attitude to the dental service makes it possible that the child and the parent will follow advice from dentist.^18^

In the present study, significant correlations were observed between subjective assessments of dental appearance and objective assessment of dental aesthetics using DAI. However, future investigations in this regard are warranted, taking into consideration other variables that may influence the orthodontic treatment demand such as rural/urban variation, proximity of dentist/orthodontist/ dental college, socioeconomic status and parental education.

## CONCLUSION

The following conclusions were drawn from the present study:

1. The correlation between dental aesthetic index and satisfaction with smile was not statistically significant.
2. A significant but weak association was present between dental aesthetic index and satisfaction with dental appearance.
3. A strong statistically significant association was present between dental aesthetic index and self-assessment of relative appearance of teeth by the subjects as compared to their face.
4. A strong statistically significant association was present between dental aesthetic index and self-assessment of dental appearance relative to classmates and friends.
5. A weak statistically significant association was present between dental aesthetic index and desire for orthodontic care.

## References

[1] Otuyemi OD, Noar JH (1996). Variability in recording and grading the
need for orthodontic treatment using handicapping malocclusion
assessment record, occlusal index and dental aesthetic index. Community Dent Oral Epidemiol.

[2] Cons NC, Jenny J, Kohout FJ (1986). DAI: The dental aesthetic index. Iowa City.

[3] Cons NC, Jenny J, Kohout FJ, Freer TJ, Eismann D (1983). Perceptions
of occlusal conditions in Australia, German Democratic Republic
and the United States of America. Int Dent J.

[4] Salzmann JA (1968). Handicapping malocclusion assessment to
establish treatment priority. Am J Orthod.

[5] Summers CJ (1971). The occlusal index: A system for identifying and
scoring occlusal disorders. Am J Orthod.

[6] Onyeaso CO, Aderinokun GA (2003). The relationship between Dental
aesthetic index (DAI) and perceptions of aesthetics, function and speech
amongst secondary school children in Ibadan, Nigeria. Int J
Paediatr Dent.

[7] Evans R, Shaw W (1987). Preliminary evaluation of an illustrated
scale for rating dental attractiveness. Eur J Orthod.

[8] Brook PH, Shaw WC (1989). The development of an index of
orthodontic treatment priority. Eur J Orthod.

[9] Otuyemi OD, Noar JH (1996). A comparison between DAI and SCAN
in estimating orthodontic treatment need. Int
Dent J.

[10] Jenny J, Cons NC (1996). Comparing and contrasting two orthodontic
indices, the Index of Orthodontic Treatment need and the Dental
Aesthetic Index. Am J Orthod Dentofacial Orthop.

[11]  Shue-Te Yeh M, Koochek AR, Vlaskalic V, Byod R, Richmond S (2000). The
relationship of two professional occlusal indexes with patient?s
perception of aesthetics, function, speech and orthodontic
treatment need. Am J Orthod Dentofacial Orthop.

[12] Ansai T, Miyazaki H, Katoh Y, Yamashita Y, Takehara T, Jenny J, Cons NC (1993). Prevalence of malocclusion of high school students of Japan
according to the Dental aesthetic index. Community Dent Oral
Epidemiol.

[13] Beglin FM, Firestone AR, Vig KW, Beck FM, Kuthy RA, Wade D (2001). A
comparison of the reliability and validity of 3 occlusal indexes
of orthodontic treatment need. Am J Orthod Dentofacial Orthop.

[14] Johnson M, Harkness M, Cowther P, Herbison P (2000). A comparison
of two methods of assessing orthodontic treatment in the mixed
dentition: DAI and IOTN. Aust Orthod J.

[15] Abdullah MS, Rock WP (2001). Assessment of orthodontic treatment
need in 5112 Malaysian using Index of orthodontic treatment
need and Dental aesthetic index indices. Community Dent Health.

[16] Lobb WK, Ismail AI, Andrews CL,  Spracklin  TE (1994). Evaluation of
orthodontic treatment using Dental Aesthetic index. Am J Orthod Dentofacial Orthop.

[17] Hamdan AM (2004). The relationship between patient, Parent and
clinician perceived need and normative orthodontic treatment
need. Eur J Orthod.

[18] Birkeland K, Boe OE, Wisth PJ (1996). Orthodontic concern among
11 year old children and their parents compared with orthodontic
treatment need assessed by Index of orthodontic treatment need. Am J Orthod Dentofacial Orthop.

[19] Espeland LV, Ivarsson K, Stenvik A (1992). A new Norwegean index
of orthodontic treatment need related to orthodontic concern
among 11 year olds and their parents. Community Dent Oral
Epidemiol.

[20] Holmes A (1992). The prevalence of orthodontic treatment need. Br J
Orthod.

[21] Gosney MB (1986). An investigation into some of the factors
influencing the desire for orthodontic treatment. Br J Orthod.

[22] Abu Alhaija ES, Al- Nimri KS, Al- Khateeb SN (2004). Orthodontic
treatment need and demand in 12 to 14 years old north Jordanian
school children. Eur J Orthod.

[23] Tulloch JF, Shaw WC, Underhill C, Smith A, Jones G, Jones M (1984). A comparison of attitudes towards orthodontic treatment in
British and American communities. Am J Orthod.

[24] Gravely JF (1990). A study of need and demand of orthodontic treatment
in two contrasting National Health Service regions. Br J Orthod.

